# Increased Obesity-Associated Circulating Levels of the Extracellular Matrix Proteins Osteopontin, Chitinase-3 Like-1 and Tenascin C Are Associated with Colon Cancer

**DOI:** 10.1371/journal.pone.0162189

**Published:** 2016-09-09

**Authors:** Victoria Catalán, Javier Gómez-Ambrosi, Amaia Rodríguez, Beatriz Ramírez, Maitane Izaguirre, José Luis Hernández-Lizoain, Jorge Baixauli, Pablo Martí, Víctor Valentí, Rafael Moncada, Camilo Silva, Javier Salvador, Gema Frühbeck

**Affiliations:** 1 Metabolic Research Laboratory, Clínica Universidad de Navarra, Pamplona, Spain; 2 CIBER Fisiopatología de la Obesidad y Nutrición (CIBEROBN), Instituto de Salud Carlos III, Pamplona, Spain; 3 Obesity and Adipobiology Group, Instituto de Investigación Sanitaria de Navarra (IdiSNA) Pamplona, Spain; 4 Department of Surgery, Clínica Universidad de Navarra, Pamplona, Spain; 5 Department of Anesthesia, Clínica Universidad de Navarra, Pamplona, Spain; 6 Department of Endocrinology & Nutrition, Clínica Universidad de Navarra, Pamplona, Spain; Dasman Diabetes Institute, KUWAIT

## Abstract

**Background:**

Excess adipose tissue represents a major risk factor for the development of colon cancer with inflammation and extracellular matrix (ECM) remodeling being proposed as plausible mechanisms. The aim of this study was to investigate whether obesity can influence circulating levels of inflammation-related extracellular matrix proteins in patients with colon cancer (CC), promoting a microenvironment favorable for tumor growth.

**Methods:**

Serum samples obtained from 79 subjects [26 lean (LN) and 53 obese (OB)] were used in the study. Enrolled subjects were further subclassified according to the established diagnostic protocol for CC (44 without CC and 35 with CC). Anthropometric measurements as well as circulating metabolites and hormones were determined. Circulating concentrations of the ECM proteins osteopontin (OPN), chitinase-3-like protein 1 (YKL-40), tenascin C (TNC) and lipocalin-2 (LCN-2) were determined by ELISA.

**Results:**

Significant differences in circulating OPN, YKL-40 and TNC concentrations between the experimental groups were observed, being significantly increased due to obesity (*P*<0.01) and colon cancer (*P*<0.05). LCN-2 levels were affected by obesity (*P*<0.05), but no differences were detected regarding the presence or not of CC. A positive association (*P*<0.05) with different inflammatory markers was also detected.

**Conclusions:**

To our knowledge, we herein show for the first time that obese patients with CC exhibit increased circulating levels of OPN, YKL-40 and TNC providing further evidence for the influence of obesity on CC development via ECM proteins, representing promising diagnostic biomarkers or target molecules for therapeutics.

## Introduction

Overweight and obesity are expanding on a worldwide level, becoming a major global health challenge [1,2]. Excess adipose tissue accumulation is associated with dysfunction of this endocrine organ, predisposing to the development of obesity-associated comorbidities including type 2 diabetes mellitus, cardiovascular diseases, dyslipidemia and respiratory problems [3]. Likewise, overweight and obesity are not only major risk factors for the development of multiple types of cancers, but have also been shown to be associated with poor prognosis and higher death rates [2,4–6]. In this sense, a robust link between obesity and increased incidence of colorectal cancer (CRC) is now evident [7]. Recent meta-analysis estimated an overall age-standardized relative risk of CRC of 1.4 for obese individuals [body mass index (BMI) ≥ 30 kg/m^2^] compared to individuals with normal BMI (BMI ≤ 25 kg/m^2^) [8] as well as a 5% increased risk of CRC per inch of waist circumference, an indicator of abdominal fat[9]. Additionally, increasing body weight and a higher percentage of body fat were significantly associated with increased CRC-related mortality after CRC diagnosis [10]. These findings have been also confirmed in rodent models [11].

To date, detailed mechanisms which mediate the obesity-driven effect on cancer development in humans are still poorly understood though chronic inflammation, the insulin/insulin growth factor (IGF)-1 axis as well as changes in adipokine signaling and in the intestinal microbiome are known to participate in cancer progression [5,12]. In this way, adipose tissue actively contributes to tumor growth by functioning as an endocrine organ and acting as an energy reservoir for embedded cancer cells [13]. Recently, it has been also suggested that tumors are able to recruit endogenous adipose stems cells to strengthen the features of the tumor microenvironment [14].

Adipocytes are surrounded by a network of extracellular matrix (ECM) proteins that not only serve as mechanical scaffold but also regulate cell functions in response to both endogenous and exogenous stimuli [15]. A chronic positive energy balance promoting obesity induces a rapid expansion of adipose tissue initiating a sequence of pathological processes including changes in the matrix composition of adipose tissue, adipose tissue fibrosis and alterations in gene and protein expression patterns [3,16]. This complex microenvironment greatly influences the metabolism and growth of tumor cells, providing a favorable area for tumorigenesis [14,15]. Importantly, inflammatory and ECM related proteins characteristic of adipose tissue from obese subjects, such as osteopontin (OPN), chitinase-3 like-1 (YKL-40), tenascin C (TNC) and lipocalin-2 (LCN-2), have also been directly implicated in tumor growth. OPN expression is markedly elevated in adipose tissue during the development of obesity [17] and abundant evidence suggests that it plays a critical role in chronic inflammatory diseases including several types of cancer [18]. YKL-40 is emerging as a new biomarker in patients with cancer [19] and increased circulating concentrations and visceral adipose tissue expression in obese patients with type 2 diabetes have been described [20]. A key molecule in tissue remodeling upregulated in obese states is TNC [21], which exhibits proinflammatory effects and also modulates cell migration and proliferation [22]. LCN-2 is a novel and versatile adipokine with a complex role in tumorigenesis. LCN-2 was reported to be expressed in neoplastic colon cells and has been found to correlate with tumor stage mainly exhibiting an important anti-inflammatory function [23]. Nevertheless, an involvement of LCN-2 in the promotion of angiogenesis and tumor progression has been also reported [24].

The molecular basis of the interactions between these ECM proteins, cancer cells and adipose tissue remains complex. Therefore, given the inflammatory state and the adipose tissue ECM remodeling that takes place in obesity as well as its relationship with colon cancer (CC), the aim of this study was to investigate whether obesity can influence circulating levels of these inflammation-related ECM proteins in patients with colon cancer promoting a microenvironment favorable for tumor growth.

## Material and Methods

### Patient selection

In order to analyze the effect of obesity and CC on the circulating levels of novel inflammation-related adipokines, blood samples from 79 subjects [26 lean (LN) and 53 obese (OB)] recruited from healthy volunteers and patients attending the Departments of Endocrinology & Nutrition and Surgery at the Clínica Universidad de Navarra were used. Patients were classified as LN or OB according to BMI (LN: BMI ≤ 25 kg/m^2^ and OB: BMI > 30 kg/m^2^). Volunteers underwent a clinical assessment including medical history, physical examination and comorbidity evaluation performed by a multidisciplinary consultation team. Enrolled subjects were further subclassified according to the established diagnostic protocol for CC [44 without colon cancer (non-CC) and 35 with colon cancer (CC)]. The CC samples were obtained from patients who underwent curative resection for primary colon carcinoma. None of the patients received preoperative and/or postoperative adjuvant chemo and/or radiotherapy at the time of sample obtaining. Clinicopathological characteristics of the subjects with CC included in the study are shown in Table 1. The control volunteers were healthy, were not on medication, and had no signs or clinical symptoms of cancer, liver alteration or type 2 diabetes mellitus. The study was approved, from an ethical and scientific standpoint, by the Hospital’s Ethical Committee responsible for research and was performed in accordance with The Code of Ethics of the World Medical Association (Declaration of Helsinki). The written informed consent of participants was obtained.

**Table 1:** Clinicopathological characteristics of patients with colon cancer.

**Gender, n**	
Male	22
Female	13
**Location of primary lesion, n**	
Transverse colon	5
Right hemicolon	13
Left hemicolon	15
Missing	2
**TNM stage, n**	
I	5
II	7
III	17
IV	4
Missing	2
**Differentiation, n**	
Well	3
Moderately	25
Poorly and undifferentiated	5
Missing	2
**Tumor size, n**	
< 5 cm	17
> 5 cm	8
Missing	10
**Lymph node status, n**	
Positive	12
Negative	23

TNM staging system; Tumor, Node, Metastases.

### Anthropometric measurements

Body weight was measured with a digital scale to the nearest0.1 kg, and height was measured to the nearest 0.1 cm with a Holstein stadiometer (Holstein Ltd., Crimes, UK). BMI was calculated as weight in kilograms divided by the square of height in meters. Body fat (BF) percentage was estimated by the CUN-BAE[25]. The waist was measured at the midway level between the margin of the lowest rib and the iliac crest.

### Laboratory procedures

Plasma samples were obtained by venipuncture after an overnight fast. Glucose was analyzed based on enzymatic spectrophotometric reactions by an automated analyzer (Hitachi Modular P800, Roche, Basel, Switzerland). Serum concentrations of triacylglycerol and free fatty acids (FFA) were measured by enzymatic methods using commercially available kits (Infinity^™^, Thermo Electron Corporation, Melbourne, Australia) [26]. High sensitivity C-reactive protein (CRP) and fibrinogen concentrations were determined as previously reported [27]. The carcinoembryonic antigen (CEA) was analyzed based on electrochemiluminescence (ECL) reactions by an automated analyzer (Cobas 8000, Roche, Basel, Switzerland). White blood cell (WBC) count was measured using an automated cell counter (Beckman Coulter, Inc., Fullerton, CA). Commercially available ELISA kits were used to assess circulating levels of OPN (R & D systems, Minneapolis, MN), YKL-40 (R & D systems), TNC (IBL International GMBH, Hamburg, Germany) and LCN-2 (R & D systems) according to the manufacturer’s instructions. The intra- and interassay coefficients of variation were: 3.2 and 5.9% for OPN; 4.6% and 6.0% for YKL-40; 4.9% and 5.4% for TNC and 3.7% and 6.5% for LCN-2, respectively.

### Statistical analysis

Data are presented as mean ± standard error of the mean (SEM). The program PS Power and Sample Size Calculations (edition 3.0.43) was used to determine the power of the study and sample size calculation. In order to identify biologically significant differences between groups we performed an estimation of a continuous response variable (plasma OPN concentrations). Based on previous similar studies of our own group the standard deviation was expected to be 16 ng/mL, thereby yielding the need to study at least 18 patients to be able to reject the null hypothesis with a 0.9 power expecting a minimum mean change of 18 ng/mL in an unpaired comparison between two groups. The type I error probability associated was 0.05. Anticipating a potential drop-out rate of individuals due to sample availability or methodological issues we decided to use at least 20 patients per group. Due to the good attendance of patients to our Departments the final experimental design included 44 volunteers without CC and 35 patients with CC. Differences in the proportion of subjects within groups regarding gender were assessed by using a contingency test (Chi-square test). Due to their non-normal distribution OPN, YKL-40 and CRP concentrations were logarithmically transformed. The normal distribution of the other variables was adequate for the use of parametric tests. Differences between groups were assessed by two-way ANOVA. Associations between two variables were computed by Pearson (r) correlation coefficient. Differences between groups adjusted for age were determined by analysis of covariance (ANCOVA). Multiple linear regression analysis was performed to evaluate the independent relationship of the studied variables. Receiver operating characteristic (ROC) curves were elaborated to establish cut-off points of the ECM protein levels that optimally predicted CC. The calculations were performed using the SPSS/Windows version 15.0 statistical package (SPSS, Chicago, IL). A *P* value < 0.05 was considered statistically significant.

## Results

### Characteristics of patients

The anthropometric and biochemical characteristics of the subjects included in the study are shown in Table 2. No differences in gender distribution between the groups was found (*P* = 0.340 and *P* = 0.137 regarding obesity and presence of CC, respectively). As expected, obese patients exhibited significantly higher (*P*<0.001) body weight, BMI, estimated BF and waist circumference compared to the lean volunteers. No differences in anthropometric measurements were detected between patients with or without CC. Circulating levels of FFA were significantly increased (*P*<0.001) in patients with CC but no differences were found concerning obesity. Increased levels of CRP were found due to obesity (*P*<0.001) and also the presence of CC (*P*<0.001). In this sense, fibrinogen concentrations were higher (*P*<0.01) in obese patients with CC compared to lean volunteers with CC and obese patients without CC. Circulating CEA levels were increased in patients with CC (*P*<0.05), but no differences were detected regarding the presence or not of obesity. Regarding the WBC, patients with CC exhibited an elevated percentage of neutrophils (*P*<0.001) together with a lower number of lymphocytes (*P*<0.001) and eosinophils (*P*<0.05). Interestingly, the number of monocytes was only significantly increased (*P*<0.05) in obese patients with CC. No differences in global WBC count were found regarding obesity.

**Table 2:** Anthropometric and biochemical characteristics of subjects included in the study.

	Lean		Obese				
	non-CC	CC	non-CC	CC	*P* Obesity	*P* Cancer	*P* Obesity x Cancer
**n (male, female)**	11 (5, 6)	15 (7, 8)	33 (20, 13)	20 (15, 5)			
**Age (years)**	54 ± 2	64 ± 3	55 ± 1	65 ± 3	0.518	**<0.001**	0.925
**Body weight (kg)**	63.9 ± 1.9	63.4 ± 7.1	84.3 ± 2.0	79.1 ± 2.6	**<0.001**	0.253	0.359
**Body mass index (kg/m**^**2**^**)**	22.9 ± 0.3	22.5 ± 0.4	30.4 ± 0.6	29.8 ± 3.2	**<0.001**	0.174	0.454
**Estimated BF %**	29.1 ± 2.0	29.0 ± 1.4	37.1 ± 1.1	34.2 ± 1.6	**<0.001**	0.379	0.365
**Waist (cm)**	84 ± 1	80 ± 1	100 ± 2	115 ± 15	**<0.001**	0.366	0.138
**Fasting glucose (mg/dL)**	101 ± 5	142 ± 12	111 ± 5	129 ± 5	0.823	**<0.001**	0.159
**Triacylglycerol (mg/dL)**	87 ± 12	116 ± 2	115 ± 13	124 ± 23	0.633	0.615	0.786
**Free fatty acids (mg/dL)**	12.7 ± 1.4	26.5 ± 2.4	15.2 ± 1.2	22.1 ± 1.6	0.644	**<0.001**	0.053
**Fibrinogen (mg/dL)**	356 ± 24	273 ± 90	299 ± 19	451 ± 41^†^^,^^‡^	0.159	0.418	**0.010**
**C-reactive protein (mg/L)**	0.07 ± 0.04	1.40 ± 0.69	1.58 ± 0.31	8.68 ± 1.60	**<0.001**	**<0.001**	0.069
**CEA (ng/mL)**	1.58 ± 0.32	2.55± 0.44	1.68 ± 0.28	8.41± 2.60	0.267	0.021	0.401
**Leucocyte (x10**^**9**^**/L)**	6.48 ± 0.84	8.12 ± 1.02	6.40 ± 0.32	7.67 ± 0.64	0.708	**0.047**	0.787
**Neutrophils (%)**	52.3 ± 3.9	71.9 ± 0.8	58.1 ± 1.8	64.8 ± 3.4	0.848	**<0.001**	0.176
**Lymphocytes (%)**	35.7 ± 4.7	19.2 ± 0.9	31.6 ± 1.6	22.5 ± 2.8	0.913	**<0.001**	0.257
**Monocytes (%)**	6.9 ± 0.6	6.1 ± 0.6	6.8 ± 0.3	9.9 ± 0.8*^,^^†^^,^^‡^	**0.011**	0.105	**0.008**
**Eosinophils (%)**	4.5 ± 0.4	2.4 ± 0.8	2.7 ± 0.3	2.4 ± 0.6	**0.116**	**0.046**	0.116
**Basophils (%)**	0.7 ± 0.1	0.3 ± 0.2	0.7 ± 0.1	0.4 ± 0.1	**0.796**	**0.025**	0.860

Data are mean ± SEM. BF, body fat; CEA, carcinoembryonic antigen;CC, colon cancer. Statistical differences were analyzed by two-way ANOVA or one-way ANOVA followed by Tukey’s *post hoc* test if an interaction was detected.

*P<0.01 vs lean without CC,

^†^*P*<0.05 vs lean with CC

^‡^*P*<0.01 vs obese without CC.

### Colon cancer and obesity increase circulating extracellular matrix protein levels

**Fig 1:**
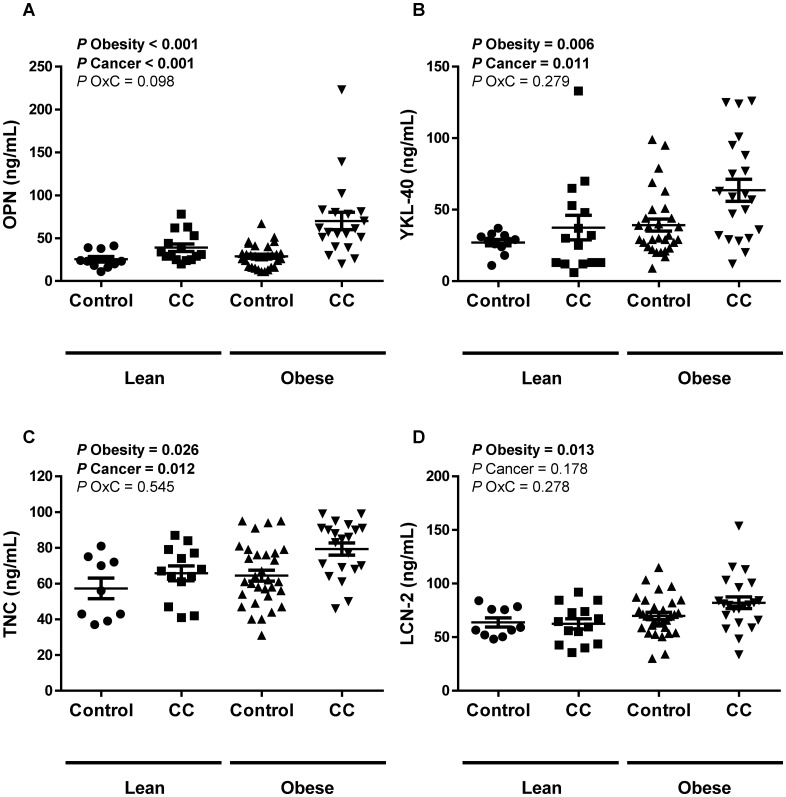
Circulating concentrations of (A) osteopontin (OPN), (B) chitinase-3-like protein 1 (YKL-40), (C) tenascin-C (TNC) and (D) lipocalin-2 (LCN-2) of lean (LN) and obese (OB) volunteers classified according the presence or not of colon cancer (CC). Differences between groups were analyzed by two-way ANOVA.

Significant differences in circulating OPN, YKL-40 and TNC concentrations between the experimental groups were observed, being significantly increased due to obesity (*P*<0.01) and colon cancer(*P*<0.05) (Fig 1). LCN-2 levels were affected by obesity (*P*<0.05), but no differences were detected regarding the presence or not of CC. Because both groups of patients with CC were significantly older, an ANCOVA with age as a covariable was performed to investigate the effect of age on plasma levels of all ECM related adipokines. We observed similar results, with OPN, YKL-40 and TNC levels being significantly increased (*P*<0.01) by obesity and cancer. The differences in LCN-2 concentrations due to obesity were also maintained after age adjustment (*P*<0.05). In this regard, plasma YKL-40, TNC and LCN-2 levels were positively associated (*P*<0.05) with BMI (Table 3). Noteworthy, a statistically significant correlation between circulating levels of OPN (*P* = 0.030), YKL-40 (*P* = 0.040) and TNC (*P*<0.001) with fibrinogen was detected as well as a positive association (*P*<0.05) of all ECM-related proteins analyzed with CRP (Table 3). Moreover, circulating levels of OPN, YKL-40 and LCN-2 were highly and positively associated (*P*<0.001) with plasma CEA levels (Table 3). Circulating levels of both, OPN (*P*<0.001) and YKL-40 (*P* = 0.026) were positively associated with the percentage of monocytes and OPN (*P* = 0.015) was also inversely correlated with the number of basophils. Multivariate linear regression analysis was also performed including age, BMI, glucose, CRP and fibrinogen in the model due to the significant association with most of the analyzed ECM-related proteins (Table 4). The analysis revealed that age, fibrinogen and CRP were significant independent determinants of circulating levels of YKL-40. BMI and CRP were significantly associated with levels of TNC while fibrinogen was associated with OPN levels and age with levels of LCN-2. Fasting glucose was not found as an independent parameter associated with the ECM-related proteins.

**Table 3:** Univariate analysis of the correlations between circulating concentrations of the analyzed markers with anthropometric and biochemical characteristics.

	OPN		YKL-40		TNC		LCN-2	
	r	*P*	r	*P*	r	*P*	r	*P*
**OPN**	-	-	0.42	**<0.001**	0.33	**0.004**	0.33	**0.005**
**YKL-40**	0.42	**<0.001**	-	-	0.24	**0.048**	0.48	**<0.001**
**TNC**	0.33	**0.004**	0.24	**0.048**	-	-	0.19	0.186
**LCN-2**	0.33	**0.005**	0.48	**<0.001**	0.19	0.186	-	-
**Age**	0.32	**0.005**	0.53	**<0.001**	0.16	0.181	0.36	**0.002**
**Body weight**	0.12	0.336	0.22	0.054	0.15	0.197	0.21	0.066
**BMI**	0.20	0.076	0.26	**0.023**	0.23	**0.044**	0.27	**0.019**
**Waist**	0.19	0.245	0.28	0.076	0.23	0.157	0.08	0.606
**Fasting glucose**	0.24	**0.004**	0.21	0.074	0.22	0.062	0.03	0.815
**Triacylglycerol**	0.12	0.317	0.25	**0.032**	-0.02	0.853	0.08	0.468
**Free fatty acids**	0.36	**<0.001**	0.33	**<0.001**	0.19	0.099	0.09	0.420
**Fibrinogen**	0.46	**0.030**	0.44	**0.040**	0.56	**<0.001**	0.29	0.162
**C-reactive protein**	0.41	**0.040**	0.58	**0.002**	0.65	**<0.001**	0.49	**0.011**
**CEA**	0.56	**<0.001**	0.46	**0.002**	0.19	0.216	0.51	**<0.001**
**Leucocytes**	0.06	0.700	0.08	0.609	0.16	0.304	0.18	0.226
**Neutrophils**	0.05	0.735	0.05	0.718	0.24	0.130	0.16	0.302
**Lymphocytes**	-0.16	0.339	-0.18	0.268	-0.26	0.101	-0.24	0.126
**Monocytes**	0.51	**<0.001**	0.35	**0.026**	0.22	0.172	0.22	0.155
**Eosinophils**	-0.03	0.836	0.22	0.168	-0.20	0.195	0.16	0.301
**Basophils**	-0.38	**0.015**	-0.29	0.069	-0.28	0.081	-0.16	0.326

CEA, carcinoembryonic antigen;OPN, osteopontin; YKL-40, chitinase 3 like 1; TNC, tenascin C; LCN-2, lipocalin-2.

**Table 4:** Multivariate linear regression analysis for the analyzed ECM-related proteins (dependent variables) including age, BMI, glucose, CRP and fibrinogen in the model.

	OPN		YKL-40		TNC		LCN-2	
Model	R^2^ = 0.558	*P* = 0.045	R^2^ = 0.881	*P*<0.001	R^2^ = 0.617	*P* = 0.018	R^2^ = 0.787	*P* = 0.004
	β	*P*	β	*P*	β	*P*	β	*P*
**Age**	0.199	0.519	0.372	**0.029**	0.198	0.498	0.866	**0.008**
**BMI**	0.399	0.109	0.232	0.062	0.458	**0.044**	0.112	0.239
**Fasting glucose**	0.328	0.226	-0.067	0.600	-0.005	0.982	0.042	0.692
**Fibrinogen**	0.735	**0.034**	0.332	**0.042**	0.121	0.638	0.144	0.577
**CRP**	-0.531	0.175	0.337	**0.046**	0.598	**0.038**	-0.129	0.913

BMI, body mass index; CRP, C-reactive protein; OPN, osteopontin; YKL-40, chitinase 3 like 1; TNC, tenascin C; LCN-2, lipocalin-2.

No differences (*P*>0.05) between male and female subjects were detected for OPN (males: 32.6 ± 2.8; females: 34.9 ± 6.0 ng/mL), TNC (males: 64.2 ± 2.9; females: 61.9±4.5 ng/mL) and LCN-2 levels (males: 73.5 ± 3.2; females: 66.7 ± 3.9 ng/mL). However, circulating concentrations of YKL-40 were significantly higher (*P* = 0.026) in males compared with females (males: 45.8 ± 5.2; females: 31.2 ± 3.5 ng/mL). Finally, we also identified a positive association between circulating levels of all the ECM-related proteins studied (Table 3).

### Predictive value of circulating extracellular matrix protein levels in the CC study population

**Fig 2:**
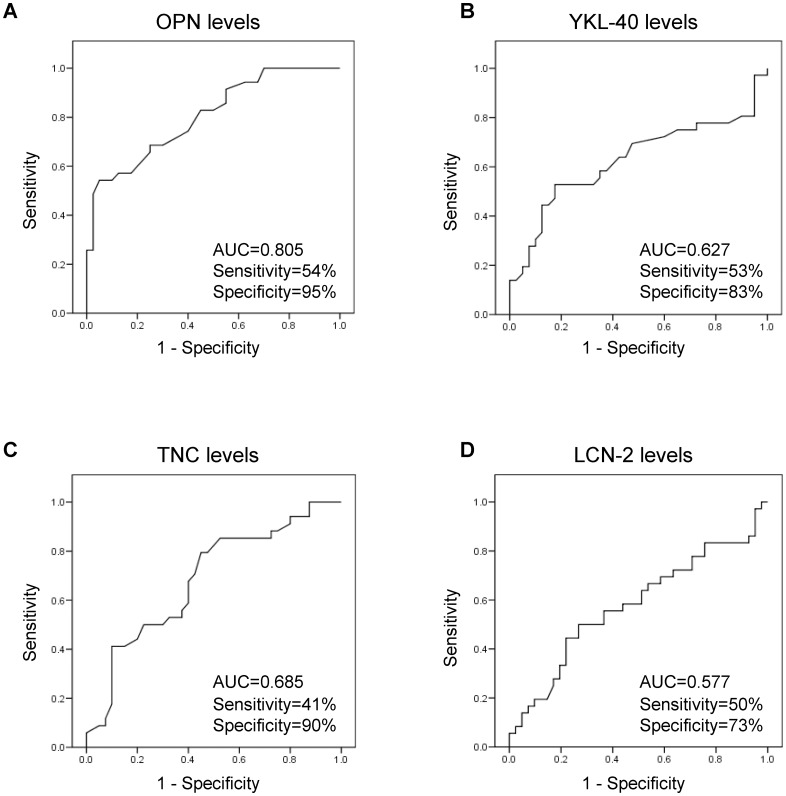
Receiver operating characteristic (ROC) curve analysis of extracellular matrix protein levels for the diagnosis of colon cancer. Area under the curve, sensitivity and specificity are indicated for each biomarker.

ROC curves were elaborated to analyze the performance of circulating levels of the analyzed extracellular matrix proteins to predict the presence of CC. Sensitivity (incidence of true positive results), specificity (incidence of true negative results), and predictive cut-off values were calculated (Fig 2). Regarding OPN levels, the area under the curve was 0.81, with the best OPN cut-off to detect CC being 48.5 ng/mL, which resulted in a sensitivity of 54% and a specificity of 95%. With respect to YKL-40 levels, the area under the curve was 0.63 and the best YKL-40 cut-off to detect CC was 45.5 ng/mL, which resulted in a sensitivity of 53% and a specificity of 83%. A TNC cut-off of 82 ng/mL resulted in a sensitivity of 41% and a specificity of 90% (area under the curve of 0.69) and finally, the cut-off of 76.1 ng/mL regarding LCN-2 levels was associated with a sensitivity of 50% and a specificity of 73% (area under the curve of 0.58).

## Conclusions

Growing evidence indicates that excess adiposity is associated with increases in cancer incidence, morbidity and mortality [4,6]. However, mechanistic insights explaining the link between obesity and increased cancer risk are notably lacking. Nowadays, it is accepted that obesity-associated chronic inflammation constitutes a contributing factor to the promotion and development of carcinogenesis [28,29]. Moreover, increasing evidence has emerged showing that the microenvironment plays a prominent role in determining tumor behavior [30]. To our knowledge, we show for the first time that obese patients with CC exhibit increased circulating levels of the ECM- and inflammatory-related proteins OPN, YKL-40 and TNC. In the obese state, the unbalanced deposition of ECM proteins in adipose tissue contributes to the formation of fibrotic depots, mechanistically related to the chronic inflammation state, which creates an optimal microenvironment for the tumor cells. Thus, altered expression of ECM proteins may represent a link between adipose tissue dysfunction in obesity and obesity-related cancer.

Recent findings have identified the involvement of OPN in CC progression with important functions in tumor infiltration and metastasis[18]. Multiple and complex mechanisms are involved in the role of OPN in cancer, including interactions with cell surface receptors, growth factor/receptor pathways, and proteases [31]. Emerging evidence underlines the potential of OPN to provide useful clinical information in the management of patients with cancer. OPN plasma levels have been found to be increased in patients with different cancers, with these higher levels being correlated with higher tumor size and staging [32]. Remarkably, we found increased OPN levels in patients with CC, which were also higher in obese patients suggesting that OPN could be a link between obesity and CC. In this line, a novel tool described for the early detection of CRC is the combination of different serum markers, including OPN[33]. An inhibition of cell proliferation and tumor colony formation by silencing of OPN has been described in human CRC cell lines highlighting its potential as molecular target for gene therapy [34]. The results of our study also demonstrate that circulating OPN significantly correlates with fibrinogen, a key pro-inflammatory biomarker upregulated in obesity also described as an independent negative prognostic marker to evaluate cancer patient risk [35]. We determined that the cut-off point for the concentration of OPN that had the best prognostic potential on the risk of CC was 48.5 ng/mL. Thus, these data suggest that for the asymptomatic obese patients, elevation of circulating OPN could be considered as a predictor of CC development. Interestingly, similar results have been obtained evaluating the role of OPN in the risk of coronary atherosclerosis [36].

YKL-40 is produced by cancer and inflammatory cells exhibiting a role in inflammation, cell proliferation and regulation of extracellular tissue remodeling and angiogenesis [37]. Specifically in CRC, high serum YKL-40 has been associated with an increased risk in subjects without co-morbidity [38]. In this regard, we observed that YKL-40 concentrations were significantly increased not only due to the presence of CC but also to obesity. Chronic inflammation is a risk factor for development of CC. In this sense, a positive association of YKL-40 with fibrinogen and CRP, well-established markers of chronic inflammation, was observed in the present study. Our finding is in accordance with a previous study where a strong association of YKL-40 with CRP was described in patients with rheumatoid arthritis [39]. In this line, circulating levels of YKL-40 increase in humans in response to increased IL-6 plasma concentrations [40]. YKL-40 as a growth factor of fibroblasts, may be contributing not only to the characteristic inflammation but also to the extensive fibrosis observed in CC [41]. A sensitivity of 53% and a specificity of 83% for YKL-40 in risk prediction of CC were found using a cut-off of 45.5 ng/mL. Although these values may not seem high, they are in fact comparable or higher to the specificity detected in other studies for YKL-40 to predict gastrointestinal cancers [42].

A class of ECM molecules called damage-associated molecular patterns (DAMPs) can directly activate the inflammatory process being rapidly released upon tisular damage [43]. TNC is a large ECM glycoprotein that belongs to this family of DAMPs and is highly expressed in tumor tissue in the majority of malignant solid tumors [44]. TNC has been proposed as a tumor marker being involved in malignant cell birth, proliferation, migration and epithelial-mesenchymal transition, angiogenesis, metastasis and evasion from therapy [45]. In this line, we observed that circulating levels of TNC were significantly increased in patients with CC being even higher in obese subjects with CC. However, although elevated TNC serum levels have been found previously in various cancers in agreement with our study, no predictive or prognostic roles have been associated to them in other studies, questioning TNC’s role as a tumor marker [46]. A possible reason resides in the influence of other comorbidities including cardiovascular or inflammatory diseases in serum TNC levels [46]. The expression pattern of TNC is dynamic, being specifically induced and tightly controlled during acute inflammation and persistently expressed in chronic inflammation [47]. In this regard, a positive association of TNC with both CRP and fibrinogen levels was detected. Data from the present study indicate that a circulating concentration over 82 ng/mL may predict the presence of CC with a specificity of 90%. However, more studies are necessary to elucidate the exact role of TNC in CC development.

LCN-2 is a component of the innate immune system that also functions as a carrier protein for small hydrophobic molecules including iron. Recently, LCN-2 has been described as an anti-inflammatory regulator not only in adipocytes but also for macrophage activation [48]. Although the role of LCN-2 in tumorigenesis has been widely investigated, its exact role in apoptosis, cell proliferation, and adhesion/invasion is not known [49]. Data from our study revealed increased levels of LCN-2 in obese patients, but no influence on LCN-2 concentration by the presence of CC. In line with our results, it has been reported that serum LCN-2 concentrations did not vary with disease stage, suggesting that the differences observed in tumor tissue are not exhibited in circulating levels of this protein [50].

Importantly, our group and others have previously shown that weight loss induced by conventional means or bariatric surgery reduces the increased circulating concentrations of these ECM proteins as well as other relevant inflammatory factors [20,27,51,52]. Moreover, bariatric and metabolic surgery are interventions with additional proven insulin-sensitizing effects [53,54] that reduce the detrimental signaling effects of the insulin/IGF1 axis, thereby exerting a positive impact on the incidence of cancer.

This study has some limitations, such as both groups of patients with CC were significantly older. The potential limitation of the older age of the CC patients in both groups has been obviated by the ANCOVA analysis with age as covariable to evaluate the effect of age on plasma levels of all ECM related adipokines. Further prospective studies to assess the involvement of these ECM proteins in the follow-up, overall, and disease-free survival of patients may contribute to better elucidate the role of these proteins in CC in the context of obesity.

Chronic inflammation and ECM remodeling both hallmark features of obesity actively contribute to the promotion of carcinogenesis by complex processes including increased cytokine secretion, immune cell infiltration or tissue injury and necrosis [55]. Inflammatory and ECM proteins are present and detectable in the systemic circulation exerting a relevant cross-talk with other mediators in the local tumor microenvironment. The fact that largely increased circulating levels of the pro-inflammatory ECM proteins OPN, YKL-40 and TNC were found in obese patients with CC provides evidence to suggest an important role of these proteins in CC development. Therefore, an influence of obesity in CC development via the ECM proteins OPN, YKL-40 and TNC can be inferred representing promising diagnostic markers or target molecules for therapeutics.

Manipulation of the inflammatory response for therapeutic benefit is an area of intense interest in cancer treatment being of great importance those therapies that selectively alter specific pathways of the immune response within the tumor microenvironment. The tumor microenvironment represents an attractive target for anticancer treatment since non-tumor cells are more likely to be genetically stable compared with tumor cells, reducing the possibility of chemotherapeutic drugs resistance. In this sense, cytokines, growth factors or components of the ECM constitute ideal targets. Importantly, the combination of biomarkers could enhance the sensitivity in the diagnosis of human cancers representing a tendency of future studies [56].
